# Occurrence of endohelminths in *Cairina moschata* (Linnaeus, 1758) of the Portel microregion, Marajó Island, Pará

**DOI:** 10.29374/2527-2179.bjvm009325

**Published:** 2026-02-20

**Authors:** Elaine Lopes de Carvalho, Yngrid da Silva dos Santos, Ricardo Luis Sousa Santana, Elane Guerreiro Giese

**Affiliations:** 1 Universidade Federal Rural da Amazônia, Belém, PA, Brazil.; 2 Laboratório de Histologia e Embriologia Animal, Instituto da Saúde e Produção Animal (LHEA), Universidade Federal Rural da Amazônia, Belém, PA, Brazil.

**Keywords:** parasites, Anatidae, Brazilian Amazon, parasitos, Anatidae, Amazônia brasileira

## Abstract

This study was conducted in northern Brazil to determine the prevalence of helminth infections in *Cairina moschata* (Linnaeus, 1758). Between August 2024 and July 2025, adult parasites were collected from the gastrointestinal tracts of 12 of these subsistence birds in the municipality of Portel, Marajó, Pará. The analyses revealed the presence of the nematodes *Ascaridia galli*, *Tetrameres* sp., *Heterakis gallinarum*, *Eucoleus contortus*, and *Capillaria cairina*; the trematodes Echinostomatidae and *Strigea* sp.*,* and various cestodes. The overall infection rate was (50%; 6/12). The most common helminths were nematodes (33.33%; 4/12), trematodes (25%; 3/12), followed by cestodes (8.33%; 1/12). These findings expand the current knowledge of the helminth fauna in muscovy ducks raised on Marajó Island and contribute to an epidemiological understanding of parasitic diseases affecting species of zootechnical importance in the Amazon region.

## Resumo

Este estudo foi conduzido no norte do Brasil para determinar a prevalência de helmintos parasitas que infectam *Cairina moschata* Linnaeus, 1758. Entre agosto de 2024 a julho de 2025, parasitos adultos foram coletados do trato gastrointestinal de 12 espécimes dessas aves de criação de subsistência no município de Portel, Marajó, Pará. As análises revelaram a presença dos nematódeos *Ascaridia galli, Tetrameres* sp.*, Heterakis gallinarum, Eucoleus contortus* e *Capillaria cairina*; os trematódeos Echinostomatidae e *Strigea* sp., e os cestódeos. O nível geral de infecção foi de (50%; 6/12) e os helmintos mais presentes foram os nematódeos (33.33%; 4/12), trematódeos (25%; 3/12), seguidos dos cestódeos (8.33%; 1/12). Esses achados ampliam o conhecimento sobre a helmintofauna de patos domésticos criados na Ilha de Marajó e contribuem para a compreensão epidemiológica das parasitoses que afetam espécies de importância zootécnica na região amazônica.

## Introduction

*Cairina moschata* (Linnaeus, 1758), also known as the Muscovy duck, is a waterfowl species native to the Americas, found from Mexico to northern Argentina, and also in the Amazon region. It lives in humid regions such as riverbanks, lakes, and swamps and is often associated with forest ecosystems (Sick, 1997).

According to Kennedy et al. (1986), the analysis of the parasitic helminth fauna of aquatic birds is considered of great importance due to the role of these birds in the dispersal of a wide range of helminth species that interact with the ecosystem. This dispersal aids in the colonization of new habitats and generates diverse parasitic communities in these birds due to their constant interaction with aquatic environments, where they forage.

Mattos Junior et al. (2008) discovered a diverse parasitic fauna in ducks from Rio de Janeiro. More recent research has confirmed the presence of the parasites *Eucoleus contortus* and *Anisakis* sp. in the esophagus of *C. moschata*, according to Carvalho et al. (2019; 2020).

Subsistence farming is common in Brazil, and the trade in live birds, eggs, and meat occurs predominantly among small producers and in open-air markets, where the hygiene and sanitary conditions of this type of farming are often unclear (Mattos Junior et al., 2008; Almeida et al., 2016).

In the northern region of Brazil, *C. moschata* is an abundant bird that is an essential component of the food supply for local communities (Carvalho et al., 2021). This study sought to identify the parasite diversity of domestic ducks in the Portel microregion, Marajó Island, with the aim of expanding knowledge about parasite biodiversity in another geographic region.

## Material and methods

### Ethical aspects

The birds were collected with authorization for activities with scientific purposes from the Ethics Committee on the Use of Animals under protocol no. 7809140122 of the Federal Rural University of the Amazon.

### Host acquisition

From August 2024 to July 2025, 12 specimens (8 males and 4 females) of *C. moschata* were analyzed. These were acquired from small subsistence farms located in the communities along the Baía de Portel, Acuti Pereira River, and Camaraipí River in the Portel microregion (Latitude -1.9365; Longitude -50.81714), Marajó Integration region in the state of Pará (Governo do Estado do Pará, 2021).

Only the respiratory and gastrointestinal tract organs were stored under refrigeration and sent to the Animal Histology and Embryology Laboratory (LHEA) for dissection. In the laboratory, the organs were separated and placed in Petri dishes with 0.9% NaCl saline solution and examined individually with a Leica ES2 stereomicroscope. The collected nematodes were washed in saline solution, fixed, and stored in A.F.A. solution (93 parts 70% ethanol, 5 parts 37% formaldehyde, and 2 parts glacial acetic acid).

### Sample processing

For optical microscopy, the nematodes were clarified in Amann's lactophenol solution and observed using a Leica DM2500 microscope with an attached camera lucida system. They were then stored in ethanol-glycerol (50 parts 70% ethanol and 50 parts pure glycerol) as per Mainenti et al. (2023). The flatworms were compressed for 72 hours and stained with Semichon acetic carmine, according to Amato and Amato (2010) and Oyarzún-Ruiz and González-Acuña (2020) and individually mounted between a slide and a coverslip using Entellan^®^ mounting medium.

Photomicrographs were obtained using a Leica DM2500 microscope with a Leica DFC310 FX camera attached as per Carvalho et al. (2024). The boards composed of images obtained with the photomicroscope were used on the Canva® platform.

### Taxonomic identification

The taxonomic studies of the helminths found were performed according to Khalil et al. (1994), Moravec (2001), Gibson et al. (2002), Anderson et al. (2009) e Bray et al. (2008).

### Parasitism index

To determine parasitism rates, prevalence (%), mean infection intensity (M_I_) and mean abundance (M_A_) were estimated, according to Bush et al. (1997).

### Specimen deposit

The material obtained is deposited in the Invertebrate Collection of the Museu Paraense Emílio Goeldi (MPEG), in Belém, Pará, Brazil, in order to ensure the documentation and preservation of the specimens for future studies. Deposit numbers for the nematode collection: MPEG.NEM 000326 – *Tetrameres* sp., MPEG.NEM 000327 – *Heterakis gallinarum*, MPEG.NEM 000328 – *Ascaridia galli*, MPEG.NEM 000330 – *Eucoleus contortus* e MPEG.NEM 000332 – *Capillaria cairina*. In the Trematode Collection: MPEG.NEM 000385 –*Athesmia heterolecithodes*, MPEG.NEM 000388 – *Strigea* sp., MPEG.NEM 000389 –*Echinostoma revolutum* e MPEG.NEM 000390 – Cyclophyllidea.

## Results

**Figure 1:**
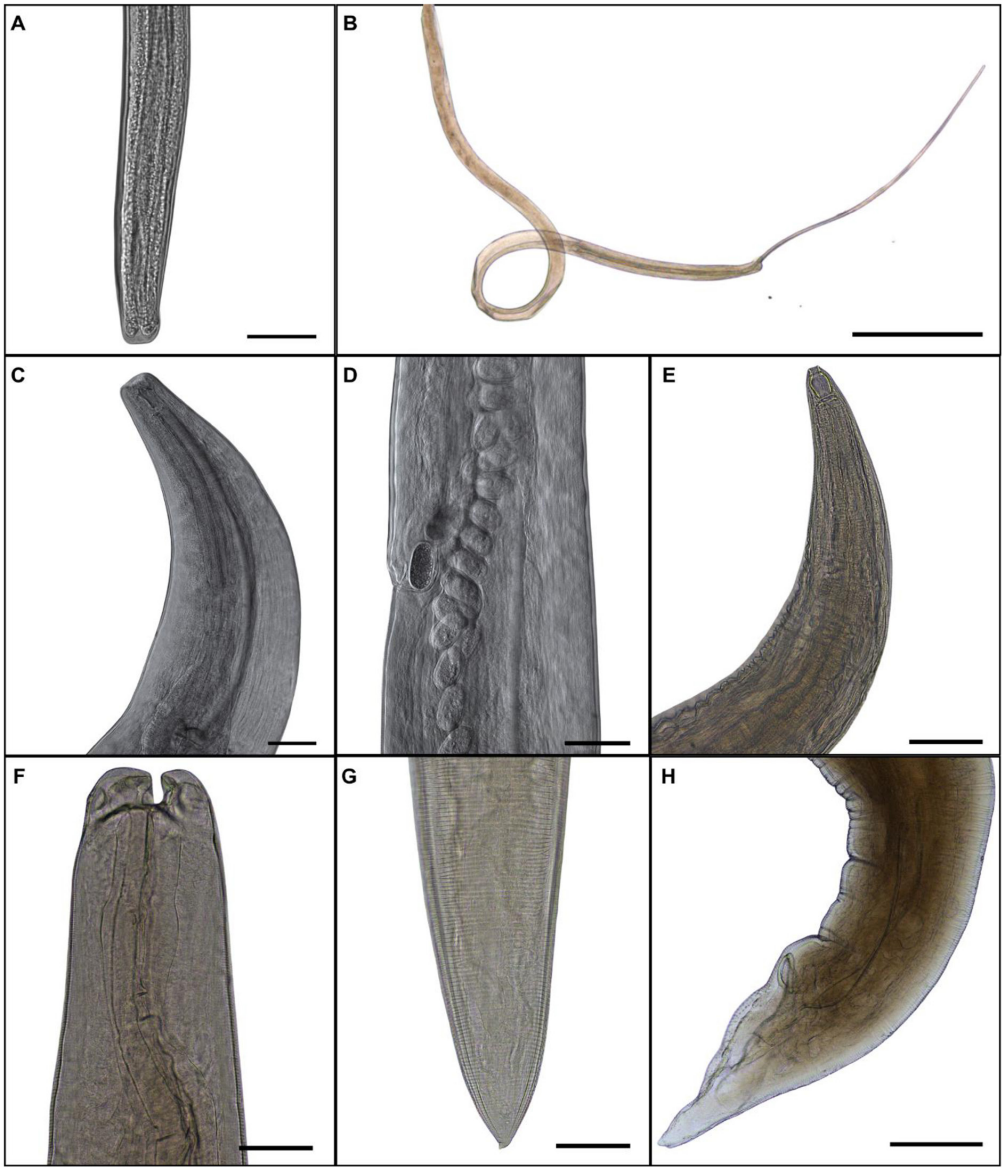
Photomicrograph of nematodes of *Cairina moschata*. A. posterior end of male *Eucoleus contortus*. Bar: 50µm; B. posterior end of male *Capillaria cairina*. Bar: 500 µm; C. *Heterakis* female. Bar: 100 µm; D. vaginal and vulvar region of gravid female of *Heterakis*. Bar: 100 µm. E. *Tetrameres* female, anterior region. Bar: 100 µm. F-G. *Ascaridia galli* female, anterior and posterior region. Bar: 200 µm. H. *Ascaridia galli* male, posterior end. Bar: 500 µm.

**Figure 2:**
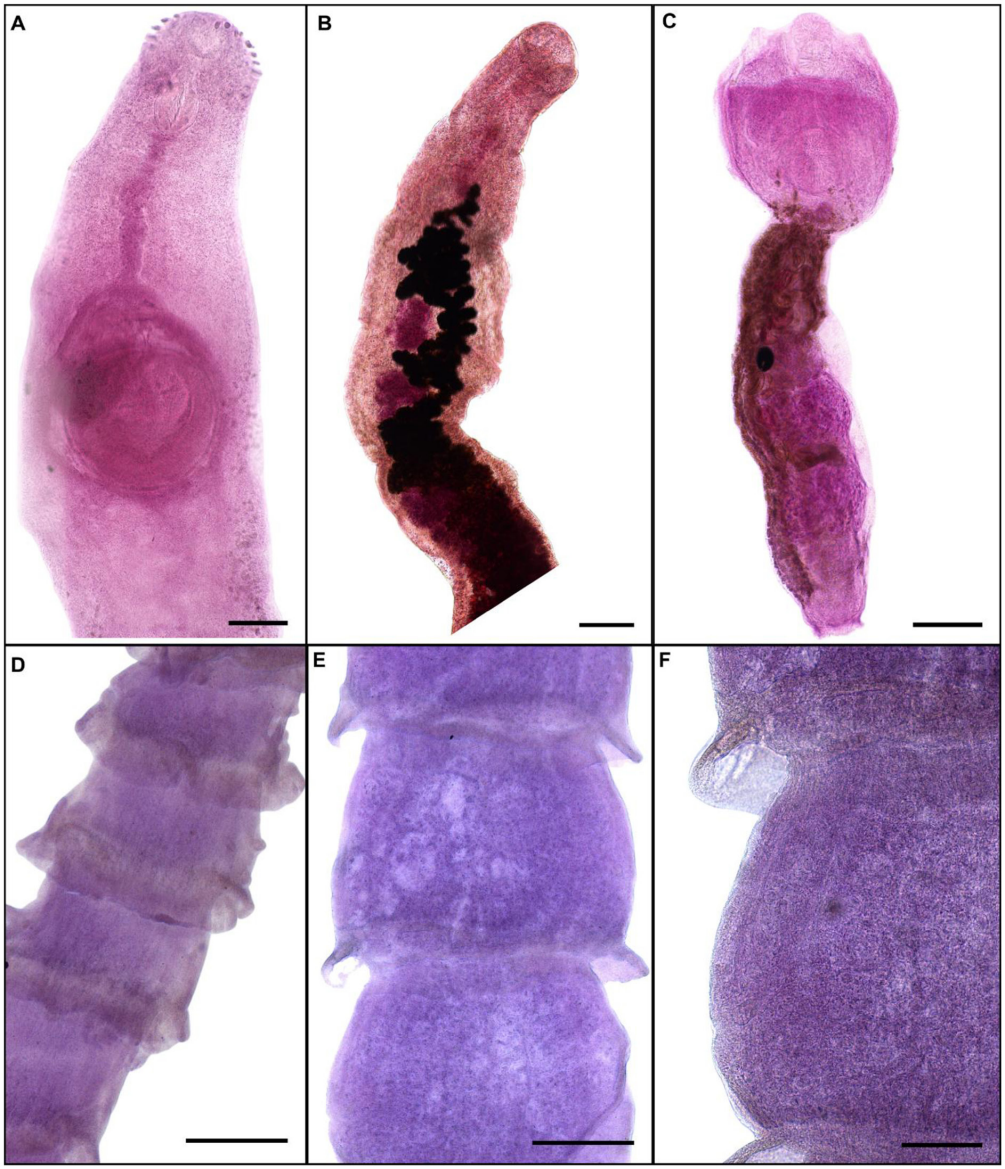
Photomicrograph of Platyhelminthes of *Cairina moschata*. A. Anterior region of Echinostomatidae. Bar: 200µm; B. *Athesmia heterolecithodes*. Bar: 200 µm; C. *Strigea* in toto. Bar: 200 µm; D-E. Cestode with immature, mature, and gravid proglottids (F). Bar: 500 µm, 500 µm, 200 µm, respectively.

Of the 12 birds examined (4 females and 8 males) 41.66% were parasitized, with a mean intensity (M_I_) of 25, a mean abundance (M_A_) of 10.41, and an infection amplitude ranging from 1 to 96 parasites per host. Data stratified according to host sex and rainfall during the collection period are presented in Table 1, while morphological descriptions are detailed below. Nematodes are shown in Figure 1, and trematodes and cestodes in Figure 2.

**Table 1:** Ecological index of parasitism of *Cairina moschata* originating from the microregion of Portel, Marajó Island, Pará, from August 2024 to July 2025.

Local	Parasite	Infection site	Number of helminths	Prevalence %	Medium intensity	Average abundance
1, 2	*Athesmia heterolecithodes*	HD, GB	8	16.66	4	0.66
1	*Heterakis gallinarum*	J	1	8.33	1	0.083
1, 3	*Ascaridia galli*	SI, J	12	33.33	3	1
3	Cyclophyllidea	SI	8	8.33	8	0.66
4	*Echinosthoma revolutum*	LI	3	8.33	3	0.25
	*Strigea* sp.	SI, LI	35	8.33	35	2.91
	*Tetrameres* sp.	P	6	8.33	6	0.5
	*Eucoleus contortus*	E	5	8.33	5	0.41
	*Capillaria cairina*	C	47	8.33	47	3.91

1 Rua Santa Mônica, Neighborhood: Cidade Nova; 2. Dr. Assis, Neighborhood: Cidade Nova; 3. Presidente Geisel, Neighborhood: Muruci; 4. Comunidade Santa Rita/Rio Camaraipi. Abbreviations: HD = hepatic ducts; J = jejunum; GB = gallbladder; SI = small intestine; LI = large intestine; P = proventriculus; E = esophagus; C = cecum*.*

In the present study, the parasite recorded was *E. contortus* (Creplin, 1839) (Gagarin, 1951), belonging to the phylum Nematoda, family Capillariidae, in *C. moschata*, infecting the esophageal mucosa of a specimen from the Santa Rita community, in the municipality of Portel, state of Pará. Medium-sized nematodes in relation to their congeners, filiform, with finely transversely striated cuticle. Cephalic region in button format. Oral aperture circular. Muscular esophagus short, narrow. Nerve ring circulating the muscular esophagus in its initial portion. Stichosome consisting of single row ; nuclei of stichocytes large and fragmented. Two wing-like pseudocoelomatic glandular cells present at esophagus-intestinal junction. Two bacillary lateral bands along the body, more numerous in females. Males spicule single, weakly sclerotized; proximal end of spicule blunt. Spicular sheath spinous.

Belonging to the same family, *Capillaria cairina*Carvalho et al. (2023), were found in the intestinal cecum of twelve of the birds examined. The nematodes were small and filiform, with delicate cuticles that were transversely striated. They exhibited a simple, dorsoventrally oriented oral opening, by 12 cephalic papillae. The nerve ring surrounded the muscular esophagus in its initial portion. The stichosome consisted of a single row of approximately 40 elongated stichocytes with transverse rings that were difficult to visualize and large stichocytes nuclei with several nucleoli. Two glandular cells were observed at the esophageal–intestinal junction. Lateral and ventral bacillary bands extended for almost the entire body length in both sexes. Males exhibited a single, heavily sclerotized spicule, with a spinous spicular sheath. The caudal end was rounded and bifurcated in ventral and dorsal views, lacking a pseudobursa and supporting two large, round ventrolateral lobes containing a pair of sessile ventrolateral pre-cloacal papillae and a terminal cloacal opening.

*Ascaridia galli* (Schrank, 1788), belonging to the class Chromadorea, order Rhabditida, and family Ascaridiidae, was recorded in *C. moschata* infecting the small intestine. The specimens were collected from birds from the Cidade Nova and Muruci neighborhoods in the municipality of Portel, Pará state. *Ascaridia galli* has an elongated, cylindrical, and somewhat semi-transparent body, yellowish-white in color, and tapering towards both ends. The entire body was enveloped by a cuticle. Triangular mouth. The dorsoventral margin appeared to be marked by a continuous ridge along the longitudinal axis of the body. Three prominent denticulate lips surrounding the mouth, with a smooth cuticle and apparently anchored to each other. The lips were of two types: one broadly elliptical in the dorsal median region and two oval in the lateroventral region.

*Heterakis gallinarum (*Schrank, 1790) Madsen, 1949, belonging to the class Chromadorea, order Rhabditida, and family Heterakidae, was recorded infecting the small intestine. The specimen was collected from a bird from the Cidade Nova neighborhood in the municipality of Portel, Pará state. Medium-sized gravid adult female. Mouth opening delimited by triradiate lips. The dorsal lip is slightly wider than the subventral lips. Each lip is covered by two triangular teeth with a spoon-shaped structure. Cephalic papillae and amphidean pores on the outer surface of the lips. Cuticle distinctly striated. The esophagus is cylindrical and slightly elongated towards the posterior end with a posterior bulb. Eggs not embryonated. However, the specific identification of this last parasite was hampered by the fact that the available material was restricted to a single fragmented female.

*Tetrameres* sp. Creplin, 1846, belonging to the class Chromadorea, order Rhabditida, and family Tetrameridae, was recorded parasitizing the proventriculus. The specimen was found in a bird from the Santa Rita community in the municipality of Portel, Pará state. Adults with marked sexual dimorphism. In this study, only females were recorded in the proventricular glands: labial region of females with papillae. At a focal level below the papillae, the well-sclerotized lining of the stoma is clearly visible. The stomatal capsule is followed by the muscular and glandular regions of the esophagus. Simple esophago-intestinal junction. The female's body is bright red when fresh, and globose. Anterior esophageal region projecting from the central globular portion of the body; posterior portion more triangular with vulva and anus projecting. No visible cuticular spines. Muscular region of the esophagus about one-quarter the length of the glandular region. Nerve ring surrounding the muscular region of the esophagus. Intestine very dark gray to black, wide, flattened, saccular, reflexed.

The species *E. revolutum* belonging to the phylum Platyhelminthes, class Trematoda, family Echinosthomatidae, was recorded parasitizing the large intestine. The parasite was found in a bird from the Santa Rita community in the municipality of Portel, Pará state. Parasite with a crown of spines in the anterior region, oral sucker and pharynx present, cirrus sac, rounded testes and ovary anterior to the anterior testes, and excretory pore in the shape of an "I".

*Athesmia heterolecithodes* (Braun, 1899) Looss, 1899, belonging to the order Plagiorchiida, family Dicrocoeliidae, and genus *Athesmia* Looss, 1899, was recorded parasitizing the gallbladder and hepatic ducts. The specimens were found in birds from the Cidade Nova neighborhood, in the municipality of Portel, state of Pará. Elongated body, smooth cuticle, and tapered extremities. Subterminal oral sucker, pre-equatorial acetabulum, and strong muscular pharynx. Long, thin intestinal ceca extending to the posterior end of the body, terminating at different levels, with the side containing the vitalarium being longer. Genital pore immediately after the cecal bifurcation. Pre-acetabular cirrus sac containing a spiral seminal vesicle. Prostatic region and cirrus poorly defined. Testicles in tandem, post-acetabular, pre-equatorial, pre-ovarian, intracecal, lobed with separate or almost contacting zones and nearly coincident fields; ovary more or less lobed, post-testicular, slightly pre-equatorial, laterally displaced, intracecal, in the field of the posterior testicle, as described by Carvalho et al. (2025a).

*Strigea* sp. Abildgaard, 1790, belonging to the order Diplostomida and family Strigeidae Railliet, 1919 was recorded parasitizing the small and large intestines. The parasite was found in a bird from the Santa Rita community in the municipality of Portel, Pará state. The description of the trematode is as follows: integumentary spines absent on the surface of the anterior part of the body. The anterior part of the body is shorter and wider. The posterior part of the body is long, slightly curved dorsally, with a smooth integument. Terminal oral sucker well developed. Ventral sucker well developed, larger than the oral sucker. Pseudosuckers are well developed, with conspicuous folds in the anterior section. Testis in tandem, bilobed, located near the posterior end of the body. Seminal vesicle long, sinuous, post-testicular, slightly overlapping the posterior testicle. Reniform ovary, pre-testicular. Yolk follicles of different sizes in both body segments. The copulatory bursa is a large enlargement. Uterus with large and numerous eggs. Terminal excretory pore.

A cestode belonging to the phylum Platyhelminthes, class Cestoda Rudolphi, 1808, and order Cyclophyllidea was recorded parasitizing the small intestine. The helminth was found in a bird from the Muruci neighborhood, in the municipality of Portel, state of Pará. Diagnosis: Cyclophyllidea, Dilepididae. Strobila small to medium-sized, dorsoventrally flattened. Scolex absent, lost during collection. Immature, mature, and gravid proglottids present. Genital pores irregularly alternate, conspicuously posterior. Genital atrium deep and narrow.

## Discussion

In Brazil, initial studies on the parasite diversity of *Cairina moschata* from Marajó Island recorded *Capillaria* sp., *E. contortus*, *Subulura* sp., larvae of *Anisakis* sp., *Contracaecum* sp., *Hysterotylacium* sp., *Raphidascaris* sp., *Eustrongylides* sp., *Syngamus* sp., *Ascocotyle* sp., and *Athesmia heterolecithodes* (Carvalho et al., 2021). They also recorded a new species of capillariid, *C. cairina*, and the trematodes *Ophthalmophagus magalhaesis* and *Typhlocoelum cucumerinum* in Santa Cruz do Arari on Marajó Island (Carvalho et al., 2023; 2025a).

According to the record of helminths in this research, it can be inferred that *E. contortus*, *C. cairina*, and *A. heterolecithodes* are common parasites of *C. moschata* (Carvalho et al., 2019; Muhairwa et al., 2007; Carvalho et al., 2023; Carvalho et al., 2025a).

*Tetrameres* were recorded by Vicente et al. (1995), Machado et al. (2006), Mattos Junior et al. (2008), and Kamil et al. (2011) in the proventriculus of *C. moschata* in Brazil and India. *Ascaridia galli* was recorded in the intestine by Alexander and McLaughlin (1997) and Muhairwa et al. (2007). In our study, the same parasite was recorded in the small intestine. *Heterakis* sp., recorded by Machado et al. (2006) in *C. moschata* in Brazil, was also identified in Portel ducks.

The occurrence of trematodes of the families Echinostomatidae, Strigeidae, and Dicrocoeliidae in domestic ducks reflects the complexity of biological cycles in these parasites, which often involve intermediate aquatic hosts, such as mollusks and fish, which are abundant in flooded environments in the Amazon region. Species belonging to the family Echinostomatidae are recognized for their zoonotic potential and are able to infect humans through the ingestion of metacercariae in undercooked fish or mollusks, which reinforces their importance in public health (Haseeb and Eveland, 2000). Strigeidae, in turn, includes species that can cause significant tissue damage in birds, compromising gastrointestinal function and, consequently, zootechnical performance.

Dicrocoeliidae, although less common in ducks, are important because they cause liver alterations, which can predispose the animals to metabolic disorders. Thus, the identification of these families in *C. moschata* highlights not only the vulnerability of these birds kept in extensive systems but also the need for continuous epidemiological surveillance to reduce impacts on production and potential risks to human health (Carvalho et al., 2025a).

The cestodes recorded in *C. moschata* are *Lateriporus biuterinus*, *Biuterina longiceps*, *Hymenolepis bisaccata*, *Hymenolepis lanceolata*, *Hymenolepis megalops*, *Hymenolepis papillata*, and *Fimbriaria fasciolaris* (Ransom, 1909), according to Ransom (1909) and Justo et al. (2017). It was not possible to perform taxonomic identification of the cestodes to the genus level, since all recovered specimens lacked a scolex, which made morphological analysis under light microscopy unfeasible. This limitation reinforces the need for new collections to obtain more complete and representative material.

When raised extensively, the birds' diet varies through the ingestion of plants, roots, small fish, snails, and insects. Supplementation is necessary if they are in the laying phase or are raised for meat, and rice, corn, cassava derivatives, sweet potato, and other food sources can be provided (Meulen and Dikken, 2003; Carvalho et al., 2020). Backues (2015) listed the main endoparasitic diseases in Anseriformes as nematodiases caused by *Cyathostoma bronchialis*, *Echinuria uncinata*, *Amidostomum* sp., and *Epimidiostomum* sp.; cestodiases; and trematodiases caused by *Cyathocotyle* sp., *Leyogonimus* sp., and *Sphaeridiotrema* sp.

Poultry farming by family farmers plays a fundamental role, both by supplementing income and by providing quality animal protein, which contributes to a healthier diet (Eiras, 2013). However, food security must be considered a priority. In this regard, when analyzing fecal samples from ducks raised in an extensive system in Colombia, Pérez et al. (2025) recorded the presence of *Capillaria* spp., *H. gallinarum*, *A. galli*, and high numbers of coccidia. These findings demonstrate that the farming system significantly influences the parasite load and the frequency of gastrointestinal helminths in domestic ducks, especially in extensive production models.

Regardless of the breeding system, whether for subsistence or commercial purposes, routine laboratory evaluation through coproparasitological examinations is essential for monitoring and controlling worm infestations in domestic ducks (Carvalho et al., 2025b; Santos et al., 2025). Studies have demonstrated the presence of endohelminth eggs, such as *Ascaridia*, Dicrocoeliidae, and *Athesmia* sp., in *Cairina moschata*, including those raised in association with *Gallus gallus domesticus* (Pérez et al., 2025).

## Conclusion

A significant diversity of helminths was found parasitizing traditionally raised *Cairina moschata* in Portel, on Marajó Island, Pará. The detection of nematodes, trematodes, and cestodes demonstrates that these birds are exposed to different parasitic cycles, reflecting the region's environmental and health conditions. The record of species such as *A. galli*, *Heterakis* sp., *E. contortus*, and *C. cairina* reinforces the importance of continuous monitoring, as these parasites can compromise zootechnical performance and animal welfare. Furthermore, characterizing the local helminth fauna contributes to understanding the epidemiological dynamics of parasitic diseases in Amazonian poultry and provides support for the development of management and health control measures aimed at subsistence farming, given the zoonotic potential of Echinotosmatidae and cestodes.
